# Non-Clinical Variables Influencing Cesarean Section Rate According to Robson Classification

**DOI:** 10.3390/medicina56040180

**Published:** 2020-04-15

**Authors:** Noemi Strambi, Flavia Sorbi, Gian Marco Bartolini, Chiara Forconi, Giovanni Sisti, Viola Seravalli, Mariarosaria Di Tommaso

**Affiliations:** 1 Department of Health Sciences, University of Florence, Obstetrics and Gynecology, Careggi University Hospital, 50134 Florence, Italy; noemi.strambi@gmail.com (N.S.); forconi.chiara@gmail.com (C.F.); mariarosaria.ditommaso@unifi.it (M.D.T.); 2 Department of Experimental, Clinical and Biomedical Sciences, University of Florence, Obstetrics and Gynecology, Careggi University Hospital, 50134 Florence, Italy; flavia.sorbi@unifi.it (F.S.); gianmarco.bartolini@gmail.com (G.M.B.); 3 Department of Obstetrics and Gynecology, New York Health and Hospitals/Lincoln, Bronx, NY 10451, USA; gsisti83@gmail.com

**Keywords:** cesarean section, labor & delivery, obstetrics, private practice, Robson classification, vaginal birth

## Abstract

*Background and Objectives*: The incidence of cesarean section (CS) has progressively increased worldwide, without any proven benefit to either the mother or the newborn. The aim of this study was to evaluate the association between CS rates and both clinical and non-clinical variables, while applying the Robson classification system. *Materials and Methods*: This is a retrospective observational study of pregnant women delivering at a tertiary care hospital between 2012 and 2017, either under public or private healthcare. The overall CS rate, and the elective and non-elective CS rate, divided by classes of Robson, were determined. The rate of vaginal deliveries and CSs was compared between the public and private setting. The distribution of incidence of non-elective CSs and their main indications were analyzed between daytime and nighttime. *Results*: 18,079 patients delivered during the study period: 69.2% delivered vaginally and 30.8% by CS. Robson class 5 was the most frequent (23.4%), followed by class 2B (16.8%). Of the 289 private practice deliveries, 59.2% were CSs. The CS rate was significantly higher in private compared to public practice: 59.2% and 30.4%, respectively (OR 3.32, 95% CI 2.62 ± 4.21). When only considering elective CSs, a statistically significant difference was found in Robson class 5 between private and public practice, with the latter having more CSs (94.2% and 83.8%, respectively (*p* = 0.046)). The rate of non-elective CS was significantly lower during nighttime than during daytime (17.2% vs. 21.5%, *p* < 0.01). During daytime, the higher incidence of CS occurred between 4:00 and 4:59 pm, and during nighttime between 9:00 and 9:59 pm. Failed induction was significantly more common as an indication to CS during daytime when compared to nighttime (*p* = 0.01). *Conclusions*: This study identified two non-clinical variables that influenced the CS rate: the type of healthcare setting (private vs. public) and the time of the day. We believe that these indications might be related more to the practitioner attitude, rather than objective delivery complications.

## 1. Introduction

Cesarean section (CS) is the most common obstetric surgery performed in the world. In recent decades, the incidence of CS has progressively increased worldwide [1,2,3]. The CS rates show wide variation among countries in the world, ranging from 0.4 to 40 percent. In Italy, the CS rate changed from 10% in 1980 to 33% in 2000, showing an increase of 196% from 1980–2000 [4]. It has been reported that subjective indications, such as non-reassuring fetal status and labor arrest disorders [5], contribute to a larger proportion in the rise of the CS rate compared to objective indications [6]. Indeed, the consistent rise in the CS rate is mainly due to non-clinical indications, such as doctors’ fear of malpractice litigation, patients’fear of labor pain, convenience for the mother in scheduling in advance the day of the delivery, and, last but not least, the misconception that CS ensures a birth with no complications [7].

Many clinicians believe that a primary CS would benefit the patient, regardless of the underlying indications [8,9,10]. On the contrary, there is recent evidence that vaginal delivery would be the best option for maternal and neonatal health [11,12]. Moreover, women undergoing CS will most likely choose to have another CS in subsequent pregnancies, leading to a further increase in the CS rate, with the well-known associated complications of repeated surgeries.

In 2014, the World Health Organization (WHO) revisited the optimal CS rate at both population and hospital level, and identified a universal classification system for CS. Additionally, WHO conducted a worldwide study to assess the association between CS and maternal and neonatal mortality [13,14]. These two studies demonstrated that, at the population level, if the CS rate rises towards 10–15%, the number of maternal and newborn deaths decreases. When the rate goes above 10–15%, there is no evidence that mortality and morbidity rates get any better. On the other hand, at the hospital level, there was no standard classification system for CS that would allow the use of CS rates to compare maternal and perinatal outcome for different facilities, cities, countries, or regions. Therefore, WHO recommended adopting the Robson classification system as a global standard for assessing, monitoring, and comparing CS rates within healthcare facilities over time, and between facilities. The classification allocates pregnant women at the time of delivery into 10 groups, which are mutually exclusive. The groups are based on five obstetric characteristics that are routinely collected in every care setting: parity, onset of labor, gestational age, fetal presentation, and number of the fetuses (Table 1).

Indeed, the Robson classification can be easily applied to evaluate the CS rates and their implications and, nowadays, the classes where CS rate should be lowered are well defined; nevertheless, we think that the Robson classification has some limitations as based only on clinical- variables. For instance, a recent Cochrane has proved that interventions on non-clinical variables are effective in reducing the number of unnecessary CSs [15].

In the present study we evaluated the association of non-clinical variables such as type of setting (private or public) and time of the day at which deliveries occurred with the CS rate in our population. These associations may impact the developing of new interventions aimed at reducing unnecessary CSs. 

## 2. Materials and Methods

This is a retrospective observational study on pregnant women delivering at Careggi University Hospital (Florence, Italy), a tertiary level hospital, from September 2012 to November 2017. At Careggi University Hospital, both private and public healthcare is offered. All of the data that were collected from electronic medical charts software in the time frame 2012–2017 were facilitated by the use of the electronic medical records software ARGOS 3.49 (Dedalus S.p.a., Florence, Italy).

We identified three main endpoints in this study in order to evaluate the association between CS rate and both clinical and non-clinical variables. The first endpoint was to determine the overall CS rate, and the elective and non-elective CS rate, divided by classes of Robson. Moreover, the incidence of the most frequent indications to CS was calculated in Robson classes 1 and 2, which are considered to be lower risk classes of CS deliveries.

The second endpoint was to compare the rate of vaginal deliveries (spontaneous and instrumental), and elective and non-elective CSs between patients who delivered in private and public setting. Private patients were defined as those whose delivery was funded by private health insurance or self-funded, whereas the public patients included women whose medical bills were completely covered by the Italian National Health Care system. 

The third endpoint was to compare the different distribution of non-elective CSs and their main indications between daytime and nighttime. We compared the non-elective CS rate between day and night time. The time of birth was categorized into six periods of time from 00:00 am to 03:59 am, from 04:00 am to 07:59 am, from 08:00 am to 11:59 am, from 12:00 pm to 03:59 pm, from 04:00 pm to 07:59 pm, and 08:00 pm to 11:59 pm. We calculated the rate of daytime CS and nighttime CS. At our institution, nightshift starts at 8 pm and ends at 8 am, while a daytime shift is from 8 am until 8 pm. We defined night-time CS as the CS performed during nightshift, and daytime CS as the CS performed during daytime shift. 

The study was exempted from IRB (Institutional Review Board) review because the data were fully anonymized and de-identified.

### Statistical Analysis

The chi-square test with continuity correction or Fisher’s exact test was used to compare the categorical variables. The results were significant for *p*-values <0.05. Data were analyzed using Graph Pad INSTAT3 software package (San Diego, CA, USA).

## 3. Results

There were 18,079 consecutive deliveries during the study period. Of these, 12,505 (69.2%) were vaginal deliveries, and 5574 (30.8%) were CSs. Of the CSs, 3022 (16.7% of total deliveries) were non-elective CS, and 2552 (14.1% of total deliveries) were elective CS. Table 2 shows the percentage of vaginal deliveries and CSs for each Robson class.

**Figure 1:**
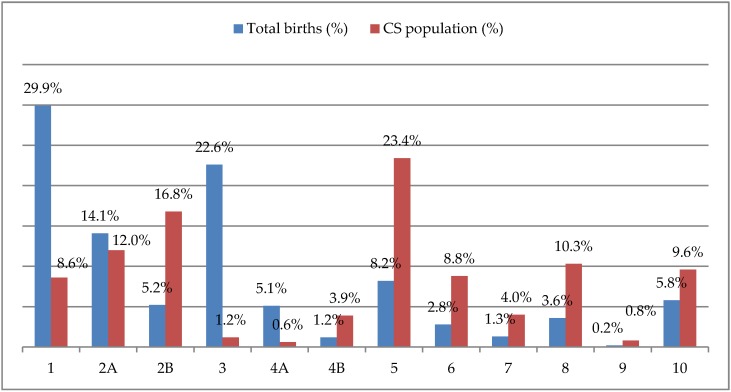
Percentages of total births and cesarean section (CS) in each Robson class.

When considering the distribution of CSs by Robson class, Robson class 5 (women with a previous uterine scar) was the most frequent (23.4%) and class 2B (nulliparous women with a single cephalic term pregnancy, delivered by CS before labor) was the second one (16.8%) (Figure 1). Singleton pregnancies with cephalic presentation of the fetus and with no uterine surgery in spontaneous labor had a low risk of CS: 8.6% in the first Robson class (nulliparous women) and 1.2% in the third one (multiparous women).

The combination of Robson class 1 and class 2, which are considered to be low risk classes for CS delivery, represented 49.1% of our study population and 37.4% of overall CSs. The main CS indications in these two low-risk classes were fetal distress (22.8%), malpresentation (21.7%), and failed induction of labor (18.5%). Other less frequent indications for CS in these classes were pelvic anomalies (6.9%) and dynamic dystocia (2.5%) (Table 3).

Private practice deliveries were 289, of which 171 were CSs (59.2%). There was a significant difference in the CS rate between private and public patients: 59.2% vs 30.4%, OR 3.32; 95% CI 2.62 ± 4.21 (Table 2). On the other hand, the rate of operative vaginal delivery was similar between private and public-private patients (10.7% vs 7.1%, OR 1.48; 95% CI 0.81 ± 2.69). 

Table 4 shows the distribution of the elective and non-elective CSs in each Robson class: the overall elective CS rate was significantly higher in the private practice setting (*p* < 0.01). In particular, when divided by Robson class, class 2B and 4B (multiparous women with a single cephalic term pregnancy, delivered by CS before labor) were the only ones to have a higher frequency of CS in the private practice group when compared to public setting (*p* < 0.01 and *p* = 0.02, respectively).

In addition, we compared the vaginal delivery and elective CS rate per class of Robson in private and non-private practice, after excluding non-elective CSs, and found a statistically significant difference for Robson class 5; in public practice, there was a lower rate of elective CS (83.8% vs 94.2%, *p* = 0.046) when compared to private setting.

Table 5 and Table 6 report the hourly distribution of vaginal birth and non-elective CS.

**Figure 2:**
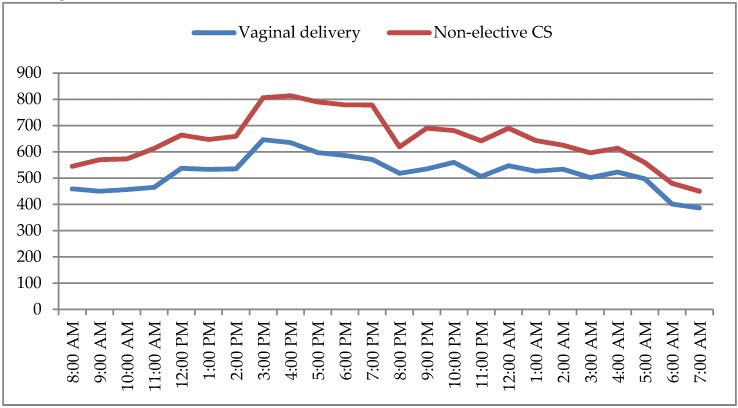
Hourly distribution of non-elective cesarean section (CS) and vaginal delivery.

The rate of non-elective CS was significant lower during nighttime than during daytime (17.2% vs. 21.5%, *p* < 0.01) (Table 7). During daytime, the lower incidence of CS was between 8:00–8:59 am and the higher between 4:00–4:59 pm; during nighttime, the higher incidence of CS was between 9:00–9:59 pm, while the lower incidence of CS was in the last period of the night shift, between 7:00–7:59 am (Figure 2).

Fetal distress was the most frequent indication to CS during both the daytime and nighttime (Table 7). 

**Figure 3:**
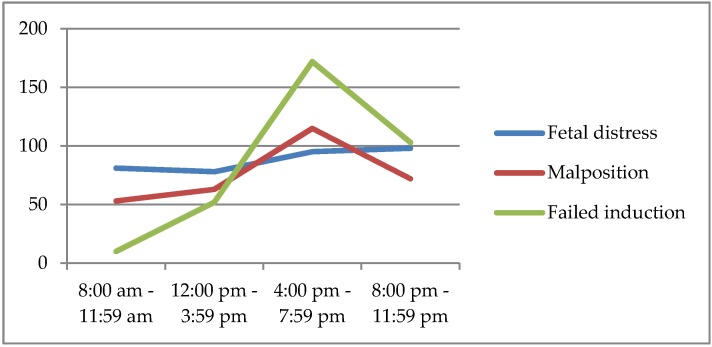
Distribution of the main indications to non-elective cesarean section (CS) during day time and nighttime.

Failed induction was significantly more common as an indication to CS during daytime as compared with nighttime (31.4% vs 24.3%; *p* = 0.01); on the other hand, there was a trend towards a higher incidence of CSs for malpresentation or arrest of labor during nighttime when compared to daytime (35.5% vs 31.0%), however the difference was not statistically significant (Table 7). It was also noticed that the peaks of malpresentation and failed induction was in the same period of the day, between 4:00 and 7:79 pm (Figure 3).

## 4. Discussion

This retrospective study showed that, in our population, the overall CS rate was 30.8% and Robson class 5 represented the most frequent class among CSs. Moreover, there was a significant difference in the overall CS rate between non-private and private settings. Vaginal deliveries and CSs had different hourly distributions, showing a difference in CS indications between daytime and nighttime.

This study not only complied with the request of WHO to adopt the Robson classification system in order to assess the CS rate at a hospital level, but it also identified the role of two non-clinical variables in the CS rate. Both aspects might allow for the creation of an effective strategy to reduce unnecessary CSs. The reasons of rising cesarean section trends worldwide are controversial, whereas it is clear that the rise in the rate of CS is not associated with reduced maternal-fetal risks or with significant improvements in perinatal outcomes [11,16,17,18].

Our first outcome was to determine the overall CS rate, and the elective and non-elective CS rate. Although the CS rate at our hospital is below the national CS rate (30.8% vs 33.7%) [19], it is still far from the WHO recommended rate of 10–15%. The non-elective CS rate alone is consistent with the average rate in Europe [14,19]. Other studies identified the main obstetric pathologies (e.g., fetal growth restriction, preeclampsia) as risk factor for primary non-elective CS and invited to consider the maternal characteristics and different assistential levels as potential bias [20,21]. Moreover, the analysis of the indicators in the first two classes of Robson, known to include the women with the lowest risk of CS, showed that fetal distress, malpresentation, and failed induction were the most frequent one. Consequently, the high CS rate of our study might be partly explained by the fact that our hospital is a tertiary care center. In our institution there is the highest number of high-risk pregnancies when compared to other hospitals of the Region and high-risk pregnancies have higher incidence of fetal distress and failed induction. In our cohort of patients, CSs primarily occurred in patients in Robson class 5 and in Robson class 2. Repeated CS is associated with a higher incidence of maternal and fetal complications when compared to vaginal birth and the first CS [22,23]. Health care providers play a decisive role in order to encourage trial of labor after cesarean (TOLAC) [24]. Currently, all of the guidelines recommend that patients with a previous CS should be thoroughly counseled during prenatal care regarding the benefits and risks of both a TOLAC and an elective repeat CS [25]. However, sometimes the fear of medico-legal litigation might influence the caregiver’s advice.

The second endpoint was to determine operative vaginal delivery and CS rate in the public and private practice setting separately and compared them. We did not find any significant difference in instrumental vaginal delivery, in contrast to the literature [26], but we found significant differences in the CS rates between private and public setting. This finding is consistent with previous studies, which reported that private patients were more likely to have a CS as compared to public patients, regardless of the Robson class [27]. Generally, the private system can be guided by financial incentives and patients’ preferences, rather than evidence-based medicine, and this has been reported as the most significant reason why the CS rate differs between public and private settings.

Our data showed that not only the overall CS rate differed significantly between the two types of settings, but also the elective CS rate, especially in Robson class 5. This indicated that women with a previous uterine scar had a higher rate of vaginal delivery in public practice than in the private setting, even if the incidence of elective CS in this class remained high in both practices. A recent Cochrane review noticed this non-clinical variable [15], where it was shown that the private practice care model might increase the repetition of CS compared to midwifery-laborist model of care [28,29]. 

In private practice, evidence-based interventions and programs to reduce both primary and repeat cesarean sections are lacking. Why providers are more apt to perform cesareans in patients that deliver in private settings is a complex issue. In our facility, antenatal care can be performed privately or through publicly funded healthcare and delivery can occur in a public or private setting, depending on patient’s choice. Regardless of the type of antenatal care, our analysis showed a significant difference in the CS trend between private and public delivery. We could argue that one of the possible explanations to the higher incidence of c-section in private practice is a lack of information regarding the benefits of TOLAC at the time of patients’ counselling. In fact, in our study, the elective CS rate among public patients of Robson class 5 was significantly lower than among private patients. Nevertheless, the level of care of a hospital, not the setting (private vs public) of the delivery, should be the main determinant in the decision on the modality of birth after a CS. The American College of Obstetricians and Gynecologists recommends that TOLAC takes place in a hospital where providers are immediately available to perform an emergency CS [30]. Our hospital is a tertiary care center and, therefore, TOLAC should be offered to all women with previous CS in the absence of clinical contraindications, regardless of whether they are private or public patients. We speculate that the increased rate in Robson class 5 CS in private delivery could be explained not only by medico-legal reasons, but also by the scheduling preferences of the provider. 

The third endpoint was to assess the hourly distribution of CS. We found that, in our study population, the CS rate was not constant over 24 hours, significantly increasing between 04:00–07:59 pm. The nighttime overall CS rate was comparable to the daytime’s, although the indications were different: the rate of non-elective CS was significant lower during nighttime than during daytime. Other studies had shown similar results [31]. 

Interestingly, non-reassuring fetal status was the most frequent indication to CS during both daytime and nighttime. On the other hand, the second most frequent indication was different: during daytime it was failed induction and during nighttime it was malpresentation. A possible explanation of the former finding is that, at our hospital, inductions are started early in the morning and usually before the end of the daytime shift an induction that has not led to birth should be labeled as failed and a CS is performed. The latter finding, a higher frequency of malpresentation as indication to c-section during nighttime, is difficult to clinically understand: perhaps it could be explained by considering physician-related variables rather than patient’s. Evidence based data show that sleep deprivation affects physicians; consequently, judgment errors may increase due to a lack of sleep during nighttime shifts [32]. 

Our work presents some strengths and limitations. One strength lies in the large number of patients that are included in the study. Moreover, the fact that our hospital includes both private and non-private delivery settings within the same institution makes the comparison between settings more reliable than in studies where private and public care occur in different hospitals. Finally, some non-clinical variables that may impact CS rate were analyzed. The limitations are mainly related to unforeseeable bias due to the retrospective nature of the study and the single-center cohort population. We did not perform a sample size calculation before beginning the study. The study did not analyze level of urgency in the definition of non-elective CS, the perinatal outcome, and pregestational or gestational pathologies. 

## 5. Conclusions

We believe that the CS rate might be reduced by monitoring CSs’ incidence and indications with the use of Robson classification, being identified by the WHO as the best global standard for studying the CS rate trend. Interestingly, our study identified two non-clinical variables that play a pivotal part in the CS rate, and that could be related more to the practitioner attitude than to the patient’s clinical condition. During nighttime, there appears to be an increase in subjective indications for CS, while, during the daytime, practitioners may be more objective. Moreover, delivering in private practice might be a risk factor for unnecessary CS. Therefore, we believe that educational interventions on the obstetricians and midwives might have an impact on the overall cesarean section rates, and that a stricter adherence to current guidelines is required for obstetricians, regardless whether the delivery occurs in public or private setting. A possible decrease in the CS rate could be achieved by reducing the impact of non-clinical variables, although further multicentric studies are necessary to better understand the complex and multifactorial origin of the increasing trend in CS rates.

## Figures and Tables

**Table 1:** The Robson classification. CS: cesarean section.

Robson Class	Description
1	Nulliparous women with a single cephalic pregnancy, ≥37 weeks gestation in spontaneous labor
2A	Nulliparous women with a single cephalic pregnancy, ≥37 weeks gestation who had labor induced
2B	Nulliparous women with a single cephalic pregnancy, ≥37 weeks gestation who were delivered by CS before labor
3	Multiparous women without a previous uterine scar, with a single cephalic pregnancy, ≥37 weeks gestation in spontaneous labor
4A	Multiparous women without a previous uterine scar, with a single cephalic pregnancy, ≥37 weeks gestation who had labor induced
4B	Multiparous women without a previous uterine scar, with a single cephalic pregnancy, ≥37 weeks gestation who were delivered by CS before labor
5	All multiparous women with at least one previous uterine scar, with a single cephalic pregnancy, ≥37 weeks gestation
6	All nulliparous women with a single breech presentation
7	All multiparous women with a single breech presentation, including women with a previous uterine scar
8	All women with multiple pregnancy, including women with a previous uterine scar
9	All women with a single pregnancy with a transverse or oblique lie, including women with a previous uterine scar
10	All women with a single cephalic pregnancy <37 weeks gestation, including women with a previous uterine scar

**Table 2:** Distribution of vaginal deliveries and cesarean sections (CSs) in each Robson class, according to government and private health insurance coverage. Chi-square statistical test was used to compare the 2 groups.

	Study Population			Non-Private Practice Population			Private Practice Population					
Robson Class	Total Births; *n*	Vaginal Delivery; *n* (%)	CS; *n* (%)	Total Births; *n*	Vaginal Delivery; *n* (%)	CS; *n* (%)	Total Births; *n*	Vaginal Delivery; *n* (%)	CS; *n* (%)	*p*-Value	OR	CI 95%
**1**	5406	4928 (91.2%)	478 (8.8%)	5363	4890 (91.2%)	473 (8.8%)	43	38 (88.4%)	5 (11.6%)	0.71	1.36	0.53 ± 3.48
**2A**	2541	1870 (73.6%)	671 (26.4%)	2517	1857 (73.8%)	660 (26.2%)	24	13 (54.2%)	11 (45.8%)	0.05	2.38	1.06 ± 5.34
**2B**	937	0	937 (100.0%)	886	0	886 (100.0%)	51	0	51 (100.0%)	-	-	-
**3**	4088	4020 (98.3%)	68 (1.7%)	4049	3981 (98.3%)	68 (1.7%)	39	39 (100.0%)	0	0.85	0.74	0.06 ± 12.10
**4A**	931	898 (96.5%)	33 (3.5%)	909	876 (96.4%)	33 (%)	22	22 (100.0%)	0	1.00	0.58	0.04 ± 9.80
**4B**	215	0	215 (100.0%)	205	0	205 (100.0%)	10	0	10 (100.0%)	-	-	-
**5**	1484	180 (12.1%)	1304 (87.9%)	1423	177 (12.4%)	1246 (87.6%)	61	3 (4.9%)	58 (95.1%)	0.11	2.75	0.85 ± 8.86
**6**	498	6 (1.2%)	492 (98.8%)	489	6 (1.2%)	483 (98.8%)	9	0	9 (100.0%)	1.00	0.26	0.01 ± 4.87
**7**	240	16 (6.7%)	224 (93.3%)	235	16 (6.8%)	219 (93.2%)	5	0	5 (100.0%)	1.00	0.83	0.04 ± 15.62
**8**	653	80 (12.3%)	573 (87.7%)	635	80 (12.6%)	555 (87.4%)	18	0	18 (100.0%)	0.15	5.36	0.32 ± 89.90
**9**	44	0	44 (100.0%)	44	0	44 (100.0%)	0	0	0	-	-	-
**10**	1042	507 (48.7%)	535 (51.3%)	1035	504 (48.7%)	531 (51.3%)	7	3 (42.9%)	4 (57.1%)	1.00	1.27	0.28 ± 5.67
**Total**	18,079	12,505 (69.2%)	5574 (30.8%)	17,790	12,387 (69.6%)	5403 (30.4%)	289	118 (40.8%)	171 (59.2%)	< 0.01	3.32	2.62 ± 4.21

**Table 3:** Indications for cesarean section (CS) in the two low risk classes (Robson class 1 and 2). “n”: number.

Indication	Non-Elective; *n*	Elective CS; *n*	Total CS; *n*	Total CS; %
Fetal distress	470	5	475	22.8%
Malpresentation	442	10	452	21.7%
Failed induction	378	8	386	18.5%
Dynamic dystocia	50	4	54	2.6%
Pelvic anomalies	27	116	143	6.9%
Maternal anxiety	10	20	30	1.4%
Cephalopelvic disproportion	17	28	45	2.2%
Fetal growth restriction	15	23	38	1.8%
Hypertension or preeclampsia	23	13	36	1.7%
Placental abruption	20	0	20	1.0%
Elderly primigravida	1	17	18	0.9%
Placenta praevia	4	19	23	1.1%
Macrosomia	2	6	8	0.4%
Gestational diabetes mellitus	6	9	15	0.7%
Oligohydramnios	8	13	21	1.0%
Maternal fever	7	0	7	0.3%
Umbilical cord prolapse	5	0	5	0.2%
Polyhydramnios	1	10	11	0.5%
Operative vaginal delivery	5	0	5	0.2%
Prolonged pregnancy	5	4	9	0.4%
Other maternal pathology	6	19	25	1.2%
Fetal pathology	11	6	17	0.8%
Others	95	148	243	11.7%
Total	1,608	478	2,086	100.0%

**Table 4:** Distribution of elective and non-elective cesarean section (CS) according to government and private health insurance coverage. Chi-square statistical test was used to compare the 2 groups.

		Non-Private Practice Population			Private Practice Population					
Robson Class	Total CS		Elective CS	Non-Elective CS	CS	Elective CS	Non-Elective CS	*p*-Value	OR	CI 95%
**1**	478	473	0	473	5	0	5	-	-	
**2A**	671	660	0	660	11	0	11	-	-	
**2B**	937	886	436 (49.2%)	450 (50.8%)	51	42 (82.4%)	9 (17.6%)	< 0,01	0.21	0.10 ± 0.43
**3**	68	68	0	68	0	0	0	-	-	
**4A**	33	33	0	33	0	0	0	-	-	
**4B**	215	205	106 (51.7%)	99 (48.3%)	10	9 (90.0%)	1 (10.0%)	0.02	0.12	0.02 ± 0.96
**5**	1304	1246	918 (73.7%)	328 (26.3%)	58	49 (84.5%)	9 (15.5%)	0.07	0.51	0.25 ± 1.06
**6**	492	483	296 (61.3%)	187 (38.7%)	9	7 (77.8%)	2 (22.2%)	0.49	0.45	0.09 ± 2.20
**7**	224	219	117 (53.4%)	102 (46.6%)	5	4 (80.0%)	1 (20.0%)	0.38	0.29	0.03 ± 2.61
**8**	573	555	327 (58.9%)	228 (41.1%)	18	14 (77.8%)	4 (22.2%)	0.15	0.41	0.13 ± 1.26
**9**	44	44	21 (47.7%)	23 (52.3%)	0	0	0	-	-	
**10**	535	531	205 (38.6%)	326 (61.4%)	4	1 (25.0%)	3 (75.0%)	1.00	1.89	0.19 ± 18.27
**Total**	5574	5403	2426 (44.9%)	2977 (55.1%)	171	126 (73.7%)	45 (26.3%)	< 0.01	0.29	0.21 ± 0.41

**Table 5:** Hourly distribution of non-elective cesarean section (CS).

	Non-Elective CS												
	Day Time												
Robson Class	8 am	9 am	10 am	11 am	12 pm	1 pm	2 pm	3 pm	4 pm	5 pm	6 pm	7 pm	Total
**1**	17	17	19	20	19	10	26	19	18	24	30	26	245
**2A**	6	8	8	20	7	20	29	37	58	60	67	72	392
2B	12	24	24	20	14	22	20	22	26	29	30	28	271
3	5	4	2	2	1	2	3	4	4	3	2	4	36
4A	0	1	0	1	4	3	2	1	1	2	6	1	22
4B	3	3	9	3	5	3	4	2	4	6	2	6	50
5	12	24	17	22	16	12	9	15	14	14	14	22	191
6	12	14	10	13	12	9	1	7	12	9	6	11	116
7	4	6	2	3	5	5	3	8	3	2	5	3	49
8	7	9	11	14	17	2	12	12	15	20	12	11	142
9	2	0	3	2	0	0	2	2	2	1	1	2	17
10	6	10	12	28	27	26	13	31	22	23	18	21	237
Total	86	120	117	148	127	114	124	160	179	193	193	207	1768
	**Nighttime**												
**Robson Class**	**8 pm**	**9 pm**	**10 pm**	**11 pm**	**12 am**	**1 am**	**2 am**	**3 am**	**4 am**	**5 am**	**6 am**	**7 am**	**Total**
1	15	14	24	20	19	24	20	25	22	14	20	16	233
2A	34	54	29	38	31	22	17	16	17	6	10	5	279
2B	17	27	24	22	22	16	8	12	10	6	12	12	188
3	2	3	2	2	3	2	5	2	1	4	2	4	32
4A	3	1	1	0	2	2	1	1	0	0	0	0	11
4B	1	6	4	5	9	4	3	4	4	5	3	2	50
5	10	14	7	15	12	18	14	11	15	11	11	8	146
6	2	7	3	6	12	7	7	4	9	2	9	5	73
7	3	6	5	8	8	5	2	5	3	3	1	5	54
8	4	12	10	6	10	5	8	7	9	8	7	4	90
9	0	0	1	2	0	0	2	0	0	0	0	1	6
10	10	11	11	12	15	12	4	7	1	3	4	2	92
Total	101	155	121	136	143	117	91	94	91	62	79	64	1254

**Table 6:** Hourly distribution of vaginal delivery.

	Vaginal Delivery												
	Day Time												
Robson Class	8 am	9 am	10 am	11 am	12 pm	1 pm	2 pm	3 pm	4 pm	5 pm	6 pm	7 pm	Total
1	197	201	212	214	223	203	206	199	241	211	201	177	2485
**2A**	20	23	31	35	49	62	86	142	143	146	143	139	1019
**2B**	-	-	-	-	-	-	-	-	-	-	-	-	
**3**	197	192	166	167	177	152	128	159	126	135	135	156	1890
**4A**	15	8	14	24	55	80	80	98	80	76	73	56	659
**4B**	-	-	-	-	-	-	-	-	-	-	-	-	-
**5**	9	9	5	8	11	8	6	8	4	7	4	7	86
**6**	0	0	1	1	0	0	0	0	1	0	0	0	3
**7**	0	3	1	1	1	0	2	1	0	2	0	1	12
**8**	2	4	4	5	5	7	2	5	9	0	5	1	49
**9**	-	-	-	-	-	-	-	-	-	-	-	-	-
**10**	19	10	22	10	16	21	25	34	31	20	25	34	267
**Total**	459	450	456	465	537	533	535	646	635	597	586	571	6470
	**Nighttime**												
**Robson Class**	**8 pm**	**9 pm**	**10 pm**	**11 pm**	**12 am**	**1 am**	**2 am**	**3 am**	**4 am**	**5 am**	**6 am**	**7 am**	**Total**
**1**	173	192	220	191	230	216	220	215	218	223	178	167	2443
**2A**	122	125	111	84	98	76	63	61	38	34	16	23	851
**2B**	-	-	-	-	-	-	-	-	-	-	-	-	-
**3**	133	146	169	175	160	189	202	180	227	198	179	172	2130
**4A**	49	33	30	26	24	17	15	16	10	11	2	6	239
**4B**	-	-	-	-	-	-	-	-	-	-	-	-	-
**5**	4	11	7	5	8	7	15	5	11	10	7	4	94
**6**	2	0	0	0	0	0	0	0	0	0	1	0	3
**7**	0	0	1	0	1	1	1	0	0	0	0	0	4
**8**	5	3	1	4	4	2	1	4	2	0	1	4	31
**9**	-	-	-	-	-	-	-	-	-	-	-	-	-
**10**	30	25	21	21	22	18	17	21	17	21	17	10	240
**Total**	518	535	560	506	547	526	534	502	523	497	401	386	6035

**Table 7:** Main indications for non-elective cesarean sections (CSs) in the two lower risk classes during daytime and nighttime. Chi-square statistical test was used to compare the 2 groups.

Indication	Non Elective CS; *n*	8:00 -11:59 am	12:00 -3:59 pm	4:00 -7:59 pm	Total Daytime	% Day Time	8:00 -11:59 pm	12:00 -3:59 am	4:00 -7:59 am	Total Nighttime	% Night Time	*p*-Value	OR (CI 95%)
**Fetal distress**	470	81	78	95	254	34.0	98	71	47	216	36.4	0.35	1.10 (0.91 ± 1.32)
**Malpresentation or no progression of the baby**	442	53	63	115	231	31.0	72	80	59	211	35.5	0.87	1.02 (0.84 ± 1.23)
**Failed induction**	378	10	52	172	234	31.4	103	27	14	144	24.3	0.01	1.52 (1.23 ± 1.87)
**Dynamic dystocia**	50	9	9	9	27	3.6	8	10	5	23	3.8	0.86	1.10 (0.63 ± 1.91)
**Total**	1340	153	202	391	746	100.0	281	188	125	594	100.0	0.01	1.17 (1.05 ± 1.31)
